# Cadmium induces mitophagy via AMP‐activated protein kinases activation in a PINK1/Parkin‐dependent manner in PC12 cells

**DOI:** 10.1111/cpr.12817

**Published:** 2020-05-12

**Authors:** Tao Wang, Qiaoping Zhu, Binbin Cao, Yan Yuan, Shuangquan Wen, Zongping Liu

**Affiliations:** ^1^ College of Veterinary Medicine Yangzhou University Yangzhou Jiangsu China; ^2^ Jiangsu Co‐innovation Center for Prevention and Control of Important Animal Infectious Diseases and Zoonoses Yangzhou Jiangsu China; ^3^ Jiangsu Key Laboratory of Zoonosis Yangzhou Jiangsu China

**Keywords:** AMPK, cadmium (Cd), mitophagy, PC12 cells, PINK1/Parkin

## Abstract

**Objectives:**

Cadmium (Cd) induces mitophagy in neuronal cells, but the underlying mechanisms remain unknown. In this study, we aimed to investigate these mechanisms.

**Materials and methods:**

The effects of Cd on the mitophagy in rat pheochromocytoma PC12 cells were detected, and the role of PINK1/Parkin pathway in Cd‐induced mitophagy was also analysed by using PINK1 siRNA. In order to explore the relationship between AMPK and PINK1/Parkin in Cd‐induced mitophagy in PC12 cells, the CRISPR‐Cas9 system was used to knock down AMPK expression.

**Results:**

The results showed that Cd treatment triggered a significant increase in mitophagosome formation and the colocalization of mitochondria and lysosomes, which was further proved by the colocalization of LC3 puncta and its receptors NDP52 or P62 with mitochondria in PC12 cells. Moreover, an accumulation of PINK1 and Parkin was found in mitochondria. Additionally, upon PINK1 knock‐down using PINK1 siRNA, Cd‐induced mitophagy was efficiently suppressed. Interestingly, chemical or genetic reversal of AMPK activation: (a) significantly inhibited the activation of mitophagy and (b) promoted NLRP3 activation by inhibiting PINK/Parkin translocation.

**Conclusions:**

These results suggest that Cd induces mitophagy via the PINK/Parkin pathway following AMPK activation in PC12 cells. Targeting the balanced activity of AMPK/PINK1/Parkin‐mediated mitophagy signalling may be a potential therapeutic approach to treat Cd‐induced neurotoxicity.

## 1 INTRODUCTION

Cadmium (Cd), an extremely toxic environmental and occupational contaminant, is present primarily in batteries, the food chain, and cigarette smoke.^1^, ^2^ Cd can severely damage several organs,^3^, ^4^ including the brain.^5^ It has been reported that Cd can cause neuronal degenerative disease, in which mitochondrial dysfunction plays a large role.^6^ The molecular mechanisms underlying Cd toxicity are multiple and complex. We have previously identified that Cd‐triggered autophagy plays an important anti‐apoptotic and anti‐senescent role in both primary rat neurons and PC12 cells.^7^, ^8^, ^9^ Furthermore, it has been demonstrated that Cd‐induced cytotoxicity in primary rat proximal tubular cells can be attributed to the inhibition of the cytosolic Ca^2+^‐dependent autophagosome‐lysosome fusion.^10^, ^11^ Accumulating evidence indicates that Cd exposure leads to mitochondrial loss in cells; however, the mechanisms underlying Cd induces mitochondrial loss during Cd‐induced neurotoxicity are not fully understood.

Mitochondria are essential for maintaining sufficient cellular ATP levels to sustain the activity of the brain.^12^ Under stressful conditions, mitochondria are selectively recruited into isolation membranes, which seal and then fuse with lysosomes to eliminate the trapped mitochondria, a process known as mitophagy. Mitophagy regulates the mitochondrial number to match metabolic demand and might also be a form of quality control to remove damaged mitochondria,^13^ and it is central to the maintenance of a healthy population of mitochondria.^14^ Furthermore, the impairment of mitophagy causes an increase in damaged mitochondria, generation of mitochondrial ROS, and release of mitochondrial DNA, which leads to overinflammation, tissue injury, and increased mortality in the host.^15^, ^16^ The most well‐known and studied mitophagy pathway, to date, has been mediated by PTEN‐inducible kinase 1 (PINK1) and Parkin, which represent a crucial amplifying mechanism that renders mitophagy more efficient.^17^ Mutations in this pathway contribute to the pathogenesis of neurodegenerative diseases.^18^ Many mechanistic studies have been conducted to explore the role of PINK1/Parkin pathway in vitro by using harsh mitochondrial toxins to activate mitophagy.^19^ Activation of PINK1/Parkin pathway promotes ubiquitination of mitochondrial outer membrane proteins and further triggers translocation of the ubiquitin‐binding receptor SQSTM1 or NDP52 to mitochondria, thus completing mitochondrial priming.^20^, ^21^, ^22^

It was recently reported that Cd induced mitochondrial loss via the overactivation of mitophagy in several different types of cells and organs.^23^, ^24^, ^25^, ^26^ However, the causative role of PINK1/Parkin‐mediated mitophagy in neurodegeneration is still under investigation. 5′‐AMP‐activated protein kinase (AMPK) has been extensively studied and highly implicated in neurons.^27^ It has been reported that AMPK and unc‐51‐like autophagy activating kinase 1 (Ulk1) play critical roles in mitophagy in primary hepatocytes and erythrocytes.^28^, ^29^, ^30^ The association of AMPK with Ulk1 regulates autophagy and phosphorylation at multiple sites.^31^ Moreover, it has been identified that AMPK could activate mitophagy to prevent heart failure via PINK1 phosphorylation.^32^

Above all, we suspected that in response to Cd‐induced mitochondrial damages, PINK1/Parkin‐mediated mitophagy was induced via AMPK phosphorylation, which could promote the clearance of damaged mitochondria, and inhibit NLPR3‐mediated inflammation in neuronal cells.

## 2 MATERIALS AND METHODS

### 2.1 Culturing of PC12 Cells

The rat pheochromocytoma (PC12) cell line was obtained from the Cell Bank of Type Culture Collection of Chinese Academy of Sciences (Shanghai, China). Cells were cultured at 37°C in a humidified 5% CO_2_ atmosphere. Cultures were maintained in antibiotic‐free RPMI‐1640 medium (Thermo Fisher) supplemented with 10% foetal bovine serum (FBS, Thermo Fisher) and 2% L‐Glutamine (Sigma‐Aldrich).

### 2.2 Transmission electron microscopy

For transmission electron microscopy (TEM) observation, after treated with 10 μM/L Cd for 6 hours, cells were collected and fixed in ice‐cold glutaraldehyde (2.5% in 0.1 mol/L cacodylate buffer, pH = 7.4) for 24 hours. Then, cells were post‐fixed with 1% osmium tetroxide for 2 hours. After dehydrated with a graded series of alcohol concentrations, the samples were rinsed in propylene oxide and impregnated with epoxy resins. Frontal sections were cut, stained with 2% uranyl acetate in 50% ethanol and lead citrate for microscopic evaluation. Finally, ultrastructure was examined with a PHILIPS CM‐120 transmission electron microscope.

### 2.3 CRISPR/Cas9 knockout cell lines

Genes were deleted in PC12 cells (Rat AMPK) by using the CRISPR‐Cas9 system. Targeting nucleotides were designed using http://crispr.mit.edu. Oligonucleotides of rat AMPK were inserted into p‐Lenti‐CRISPR‐V2 vector. The individual constructs were then subjected to transfection of HEK293T cells followed by lentivirus packaging. Transfection was performed with Optimum‐Minimum Essential Medium maintained with Lipofectamine 3000 (Thermo Fisher). All cells were incubated for 24 hours. Finally, a stable population integrated of transgene was obtained monoclonally.

### 2.4 RNA interference of PINK1

Cells (8 × 10^4^) were transfected with either 100 nmol/L PINK1‐targeting small interfering RNA (Santa Cruz) or control nonspecific siRNA (sc37007, Santa Cruz). 48 hours following transfection, the cells were exposed 10 μM/L Cd for 6 hours. Then, cells were collected and processed for immunoblotting and immunofluorescence microscopy of mitochondria‐containing autophagosomes.

### 2.5 Antibodies

Rabbit monoclonal anti‐LC3, P62, Tom20, PINK1, Parkin antibody and polyclonal antibody NDP52, beclin1, COXⅣ, GAPDH were purchased from AbCam company. Rabbit monoclonal anti‐AMPKα, phos‐AMPKα, NLRP3 and cleaved‐Caspase1 antibody were purchased from CST company. Donkey antirabbit IgG‐Alexa Fluor‐488 was purchased from Thermo Fisher.

### 2.6 Western blot

Cells were washed with PBS, lysed in RIPA buffer on ice. Supersonic lysates were determined by BCA protein assay. Protein samples (20‐80 μg) were loaded on 10%‐12% gradient polyacrylamide gels (Bio‐Rad) for Western blotting as described previously. All primary antibodies were diluted at 1:1000, the corresponding HRP‐conjugated secondary antibodies were diluted at 1:5000.

### 2.7 Immunofluorescence microscopy

To visualize mitochondria containing autolysosome formation, cells were inoculated onto 35 mm ibidi‐treated glass bottom dishes (ibidi GmbH, Germany). After treated with 75 nmol/L LysoTracker Red (Beyotime) and 200 nmol/L MitoTracker Green (Beyotime), the colocalization of lysosome with mitochondria was resolved by using laser scanning confocal microscopy (TCS SP8 STED, Leica Corporation). To further assess colocalization of proteins, cells were seeded onto coverslips (Shi‐Tai Company) overnight and fixed in methyl alcohol at −20°C for 20 minutes. Blocking and permeabilizing were performed in 5% BSA and 0.3% Triton in PBS for 1 hour at room temperature. Coverslips were then incubated with primary antibodies overnight at 4°C. Coverslips were then rinsed with PBS for 3 times, then incubated with fluorescent secondary antibodies at room temperature for 1 hour, DAPI (Beyotime) was used to visualize the nuclei. All coverslips were rinsed with PBS for 3 times and then mounted onto microscope slides. Samples were visualized with a confocal fluorescence microscope (TCS SP8 STED, Leica Corporation).

### 2.8 Statistical analysis

Statistics were determined by using the IBM SPSS Statistics 25. All error bars in the graphs represent standard deviation. Statistical comparison of mean values between groups was assessed using one‐way ANOVA analysis of Student's t test. The levels of significance were set at *P* < .05 and *P* < .01.

## 3 RESULTS

### 3.1 Cd induces mitophagy in PC12 cells

**FIGURE 1:**
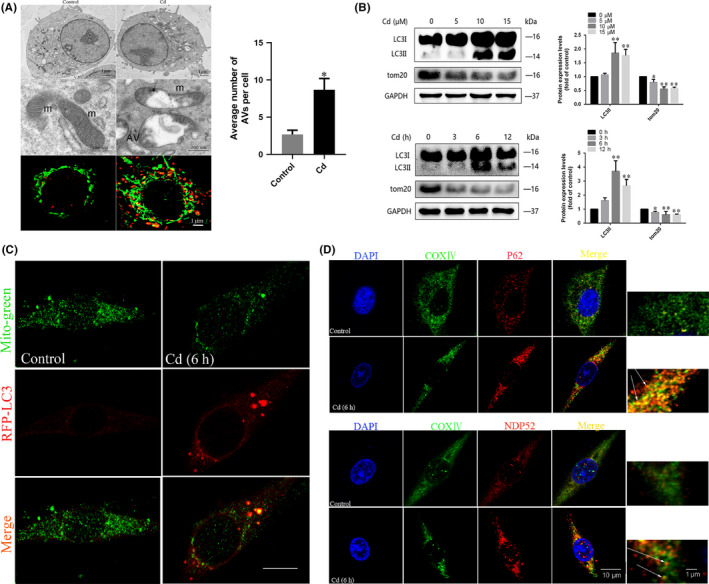
Cd induces mitophagy in PC12 cells. A, TEM analysis of autophagosomes containing mitochondria damaged by Cd (black arrows); average number of AVs (autophagosomes and autolysosomes) quantified per cell; m, mitochondrial; AV, autophagic vacuoles. To examine the connection between mitochondria and autolysosomes, the colocalization of LysoTracker Red with MitoTracker Green in PC12 cells was observed by laser scanning confocal microscopy. Red, LysoTracker Red; green, MitoTracker Green; gold, merge. The gold puncta were considered as autophagosomes containing mitochondria and counted. B, Representative immunoblots and quantification analysis of LC3 and TOM20 in Cd‐treated PC12 cells. GAPDH was used as the internal control. C, Mitochondrial localization of LC3 in PC12 cells increased upon Cd treatment, as indicated by confocal scanning microscopy. Red, RFP‐LC3; green, MitoTracker green (scale bars: 10 μm). D, The LC3 adapters P62 and NDP52 were recruited to mitochondria in PC12 cells upon Cd treatment. The colocalization of P62 with the mitochondrial marker COX IV in PC12 cells was observed by laser scanning confocal microscopy. Red, P62; green, COX IV; gold, merge. The colocalization of NDP52 with COX IV in PC12 cells was observed by laser scanning confocal microscopy. Red, NDP52; green, COX IV; gold, merge. The orange‐yellow puncta were considered as mitochondria containing NDP52 and counted. A minimum of 50 cells were analysed for each experiment. (*P < .05, **P < .01 versus control)

To determine the effects of Cd on mitophagy in PC12 cells, the morphology of mitochondria was characterized by TEM and confocal fluorescence microscopy. As shown in Figure 1A, the cells in Cd group exhibited conspicuous mitochondrial degradation compared with the control cells. Furthermore, the colocalization of mitochondria (labelled with MitoTracker Green) and lysosomes (labeled with LysoTracker Red) was observed. Treatment of cells with 10 μmol/L Cd for 6 hours triggered the colocalization of mitochondria and lysosomes (Figure 1A). We further evaluated the expression of key proteins related to Cd‐induced mitophagy, which may contribute to mitochondrial degradation. As shown in Figure 1B, Cd induced a significant dose‐dependent increase of LC3‐II exopression and decrease of TOM20 expression in PC12 cells. This transformation was verified by time course analysis by Western blot. Moreover, Cd significantly increased the colocalization of LC3 puncta with mitochondria (Figure 1C). Several autophagy receptors have been reported to play important roles in mitophagy.^23^, ^33^ To determine whether P62 and NDP52 are directly recruited to phospho‐ubiquitin signals on mitochondria, we examined the colocalization of the mitochondrial marker COX IV with P62 and NDP52. As shown in the results, both P62 and NDP52 were transported to mitochondria in Cd‐treated PC12 cells (Figure 1D).

### 3.2 Cd‐induced mitophagy is PINK1/Parkin‐dependent in PC12 cells

**FIGURE 2:**
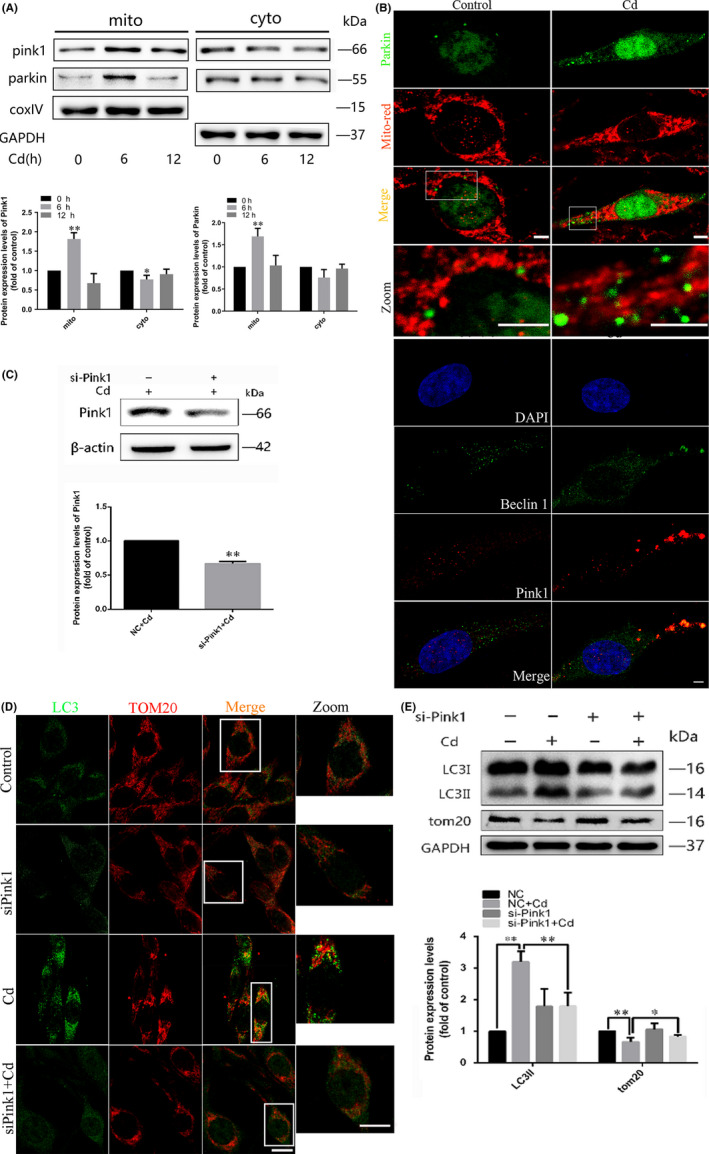
The PINK1/Parkin pathway is involved in Cd‐induced mitophagy in PC12 cells. A, Western blot analysis of PINK1 and Parkin expression in mitochondria and cytoplasm in PC12 cells after Cd exposure. GAPDH and COX IV were used as loading controls. B, Confocal scanning microscopy images of PC12 cells transfected with a rat Parkin‐GFP fusion protein and labelled with MitoTracker Red. Increased mitochondrial localization of Parkin is observed. Colocalization of Beclin1 immunofluorescence and MitoTracker Red fluorescence was also assessed by confocal microscopy analysis. Red, PINK1; green, Beclin1; gold, merge. C, A representative immunoblot of PINK1 protein levels in PC12 cells following PINK1 knock‐down using a commercial si‐PINK1 vector. β‐actin was used as the internal control. D, The colocalization of LC3 dots with TOM20 in PC12 cells following PINK1 knock‐down with si‐PINK1 and Cd treatment indicates mitochondrion containing autophagosome formations. Red, TOM20; green, LC3; gold, merge. The golden puncta were considered as mitochondrion containing autophagosomes and counted. E, LC3 and TOM20 levels in PC12 cells cultured with Cd in the presence or absence of si‐PINK1 were analysed by Western blot. GAPDH was used as the loading control. (scale bars: 1 μm, *P < .05, **P < .01)

PINK1/Parkin signalling has been reported to play an important part in mitophagy. To study the role of the PINK1/Parkin pathway in Cd‐induced mitophagy, we examined the effects of Cd on cytoplasmic and mitochondrial PINK and Parkin levels. Cd provoked an increase in mitochondrial and a decrease in cytoplasmic PINK1 and Parkin protein levels (Figure 2A), which indicated that these factors were translocated from cytoplasm to mitochondria when Cd stimulation happens. Furthermore, the Cd‐induced colocalization of mitochondrial PINK1 and Parkin was observed most obvious at 6 hours, which was consistent with the potential colocalization of PINK1 with both mitochondria and Beclin1 (Figure 2B). To further address whether Cd‐induced mitophagy was PINK1/Parkin‐dependent, PINK1 was silenced by using siRNA. As expected, the Cd‐induced increase in PINK1 expression was reversed by si‐PINK1 (Figure 2C). Particularly, PINK1 knock‐down significantly blocked the increase in colocalization of LC3 and TOM20 following exposure to 10 μM Cd for 6 h (Figure 2D). Moreover, the LC3‐II/GAPDH ratio and the expression of TOM20 was also altered by siPINK1 (Figure 2E), which further confirmed the involvement of the PINK1/Parkin pathway in Cd‐induced mitophagy in PC12 cells.

### 3.3 AMPK activation is involved in the maintenance of mitophagy in response to Cd exposure

**FIGURE 3:**
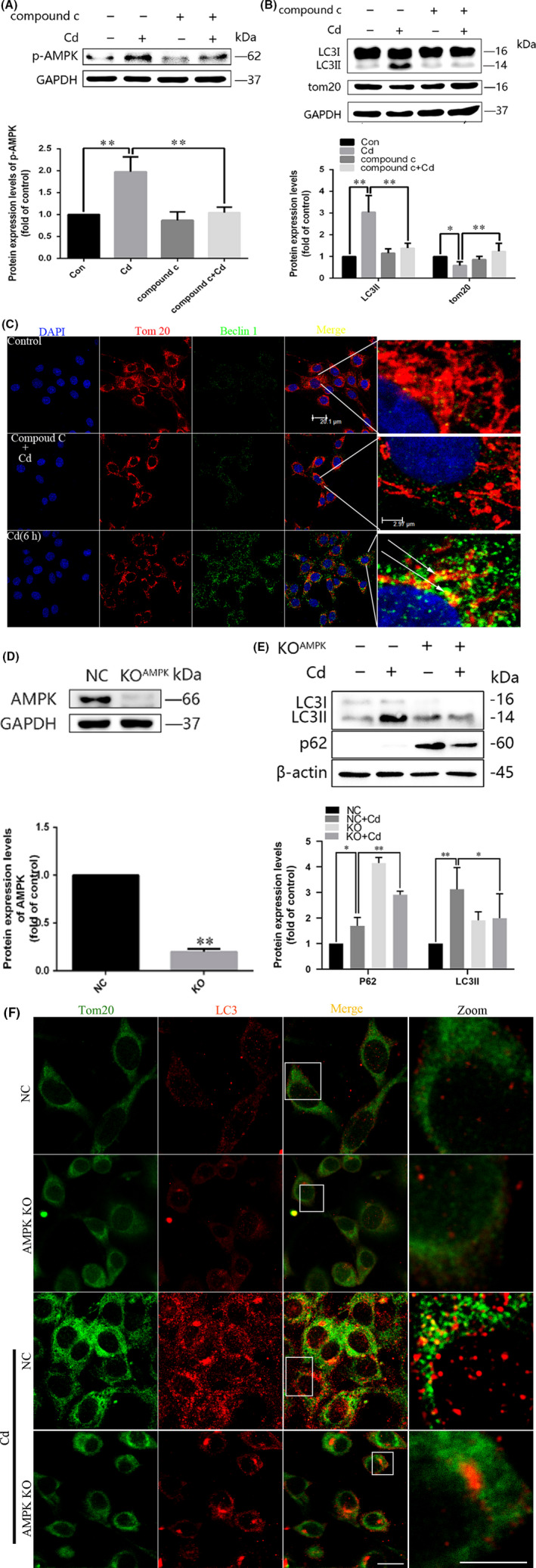
Pharmacological inhibition of AMPK activation decreases the mitochondrial localization of Beclin1 upon Cd treatment. A and B, Representative immunoblots and quantification analysis of phosphorylated AMPK (p‐AMPK), LC3, and TOM20 in Cd‐treated PC12 cells in the presence or absence of Compound C. C, After treatment with 10 μmol/L Cd for 6 h with or without Compound C, the cells were stained for Beclin1 and TOM20 and analysed by confocal microscopy. Red, TOM20; green, Beclin1. D, The AMPK gene was knocked out in PC12 cells using the CRISPR‐Cas9 system. Control cells and AMPK knockout cells were treated with Cd for 6 h, and AMPK levels were measured by Western blot. E, Effects of treatment with 10 μM Cd on LC3‐II and p62 protein levels, as measured by Western blot in AMPK knockout cells. F, The colocalization of LC3 dots with TOM20 was analysed in AMPK knockout cells following Cd treatment to visualize the mitochondrion containing autophagosome formations. Red, TOM20; green, LC3; gold, merge. (scale bars: 10 μm, *P < .05, **P < .01)

The activation of AMPK and its contributions to the initiation of mitophagy have been studied for many years.^32^ Our previous work showed that Cd triggers AMPK activation in PC12 cells.^34^ However, the role of AMPK in the activation of mitophagy during Cd exposure has not been well understood. To confirm whether the reduced activity of AMPK suppresses Cd‐induced mitophagy, we examined LC3‐II and TOM20 levels in Cd‐treated PC12 cells in the presence or absence of the AMPK phosphorylation inhibitor Compound C. As shown in Figure 3A,B, Compound C inhibited the activation of AMPK and further reduced the protein levels of LC3‐II while increasing the expression of TOM20. We then analysed whether the translocation of Beclin1 to mitochondria could be blocked by AMPK inhibition, as shown in Figure 3C, the colocalization of Beclin1 and TOM20 was inhibited by the pretreatment of Compound C. To confirm the conclusion, AMPK knockout cell lines were prepared by using the CRISPR‐Cas9 system. As shown in the results, gene defect of AMPK decreased the expression of AMPK (Figure 3D) and further increased LC3‐II expression while leading to P62 accumulation (Figure 3E). Further more, the immunofluorescence assay showed that disruption of AMPK function blocked the colocalization of TOM20 and LC3 induced by Cd (Figure 3F).

### 3.4 AMPK‐mediated mitophagy induced by Cd is PINK1/Parkin‐dependent

**FIGURE 4:**
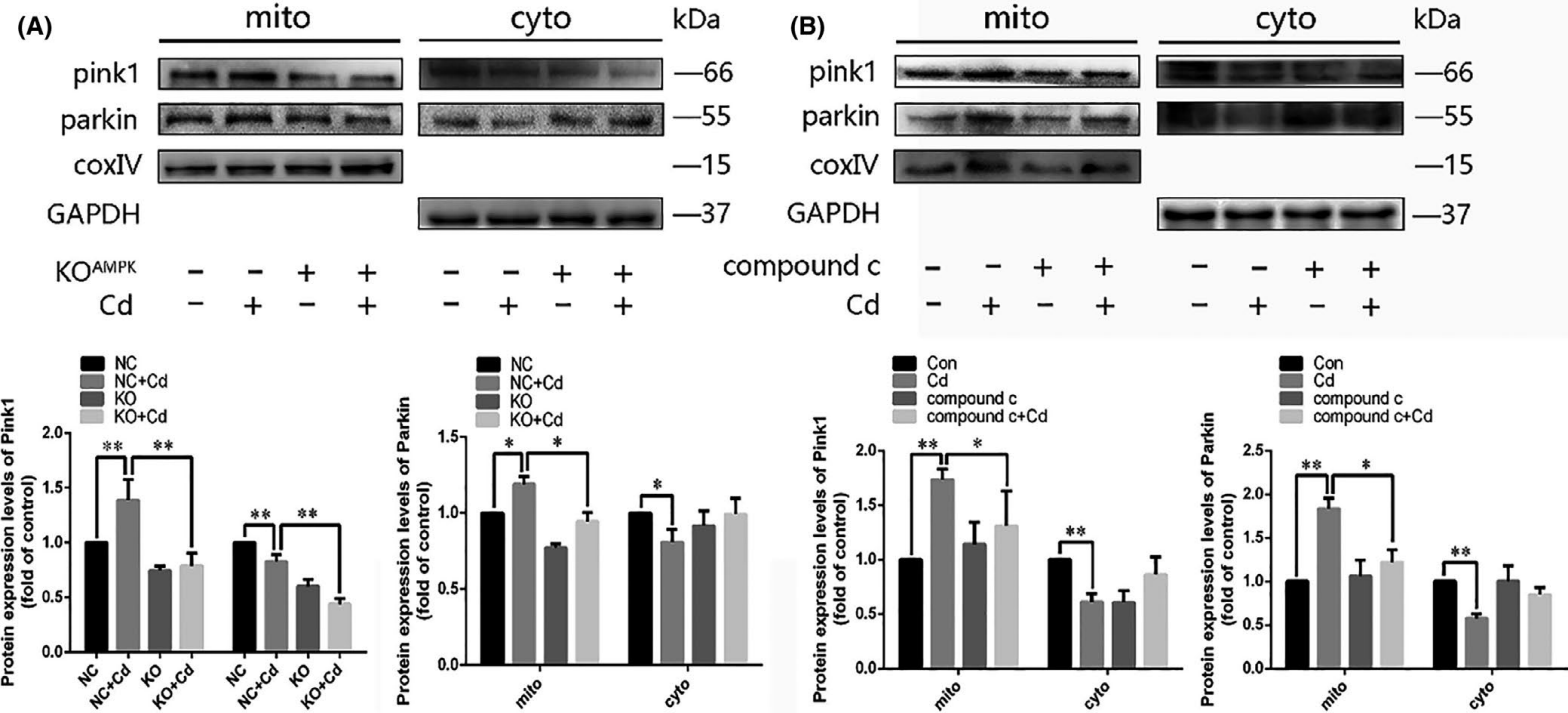
Disruption of AMPK function blocked the PINK1/Parkin pathway in Cd‐treated PC12 cells. A and B, Disruption of AMPK function in PC12 cells was achieved by a genetic mutation or chemical inhibition. After Cd treatment, mitochondrial and cytoplasmic PINK1 and Parkin levels were analysed by Western blot. The results were quantified and analysed by Image J software. (*P < .05, **P < .01)

We next explored the relationship between AMPK activation and the PINK1/Parkin pathway in Cd‐induced mitophagy. The translocation of PINK1 and Parkin from the cytoplasm to mitochondria was prevented when AMPK function was disrupted by a genetic mutation or a chemical inhibitor (Figure 4A,B). Collectively, these experiments demonstrate that AMPK plays a pivotal role in Cd‐induced mitophagy in PC12 cells, and its effects are mediated by the PINK1/Parkin signalling pathway.

### 3.5 Inhibition of mitophagy increases NLRP3 activation in response to Cd treatment in PC12 cells

**FIGURE 5:**
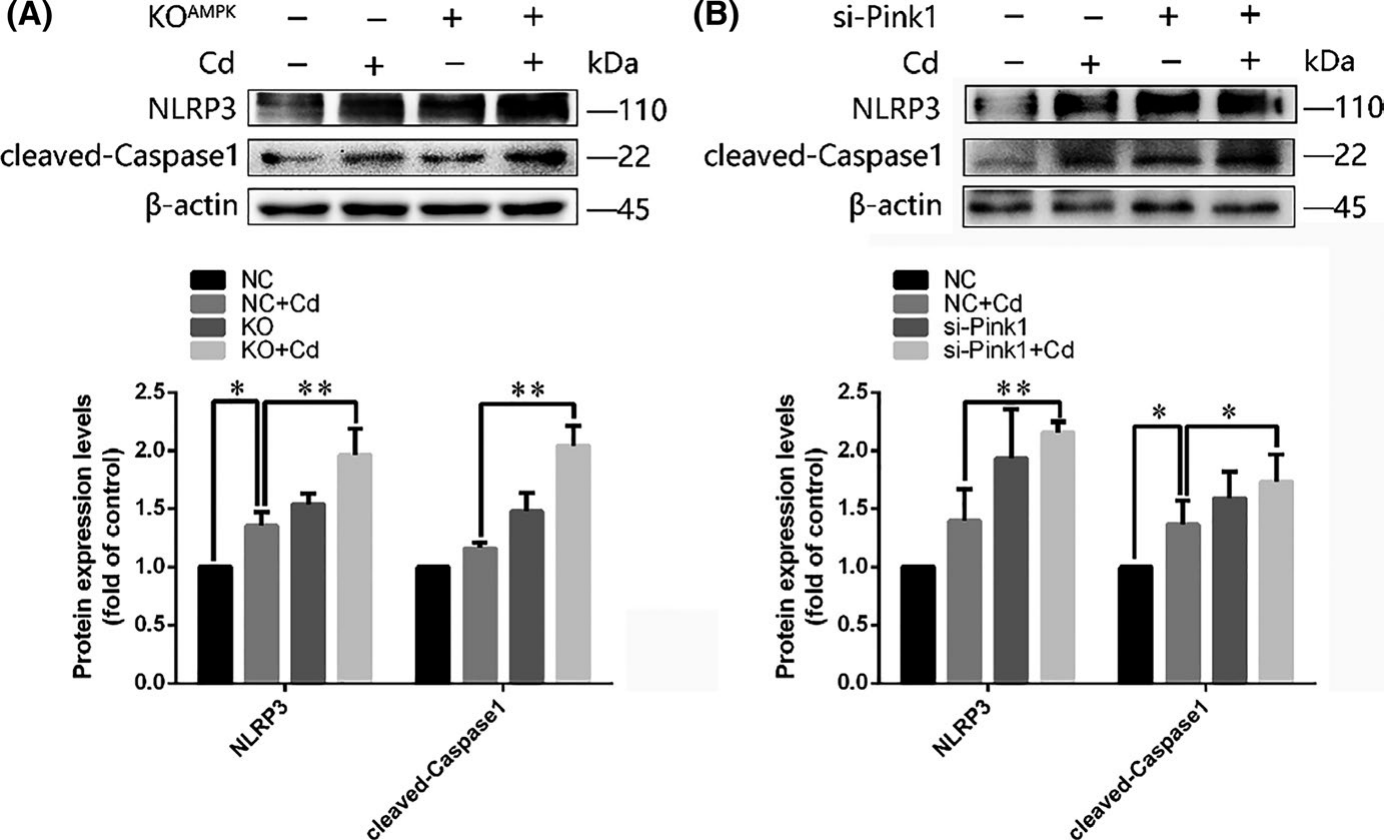
AMPK knockout and PINK1 knock‐down increased Cd‐induced NLRP3/Caspase1 activation in PC12 cells. PC12 cells with (A) AMPK knockout or (B) si‐PINK1 treatment were cultured with Cd for 6 h. Representative immunoblots and quantification analysis results of NLRP3 expression and Caspase1 cleavage are shown. (*P < .05, **P < .01)

Previous studies have reported that prolonged NLRP3 inflammasome activation was suppressed upon mitophagy‐induced clearance of damaged mitochondria in macrophages. To investigate the involvement of AMPK/PINK1‐mediated mitophagy in Cd‐triggered NLRP3 activation in neuronal cells, we examined the effects of AMPK on the activation of NLRP3 and Caspase1 in Cd‐treated PC12 cells. In the presence of Cd, AMPK defect enhanced the levels of NLRP3 expression and Caspase1 activation (Figure 5A). To explore whether this regulation is PINK1‐dependent, PINK1 expression was blocked by si‐PINK1. As shown in Figure 5B, si‐PINK1 promoted NLRP3/Caspase1 activation, which indicated that AMPK/PINK1‐mediated mitophagy may suppress Cd‐induced NLRP3 inflammasome activation in PC12 cells.

## 4 DISCUSSION

Mitochondrial dysfunction has been implicated in the process of neurodegeneration prominent in diseases, such as Parkinson's disease.^35^ Accumulating data in different cell types have suggested that mitophagy may provide critical protection against both intrinsic and environmental stresses. The molecular mechanism underlying Cd‐triggered mitophagy has been revealed by many studies. Specifically, PINK1/Parkin‐mediated mitophagy was observed in kidneys of mice exposed to Cd.^24^ However, the precise mechanisms underlying these effects in neuronal cells remain largely unknown. The results from our current study have indicated that PINK1/Parkin plays an important role in Cd‐induced mitophagy via AMPK pathway in PC12 cells. Furthermore, mitophagy may protect cells from Cd‐triggered NLRP3‐mediated inflammation.

Mitophagy occurs under various stress conditions and has been widely observed in multiple mitochondrial dysfunction‐induced diseases.^23^, ^36^, ^37^ An accumulating body of evidence indicates that mitophagy governs the mitochondrial quality control and cell fate. Here, we found that mitophagy was induced in PC12 cells at the early stage of Cd treatment, at which moment little cell death occurred. According to our results, Cd‐induced mitophagy was identified by the colocalization of mitochondria with lysosomes and the detection of mitochondrion containing autolysosome formations. Moreover, Cd increased the colocalization of LC3 and TOM20. All of these results suggest that mitophagy was induced by Cd as a result of lysosomal degradation in PC12 cells.

It has been found that several main signalling pathways are involved in the regulation of mitophagy under stress. The BH3‐only Bnip3 binds to the dynamin Opa1 to promote mitochondrial fragmentation and apoptosis by distinct mechanisms. LC3 interacts with Bnip3 to selectively digest the endoplasmic reticulum and mitochondria via autophagy. Recently, a Parkin‐independent role of PINK1 in mitophagy has been uncovered. In this pathway, PINK1 recruits P62 and NDP52 on the mitochondria, which subsequently recruit autophagy regulators to initiate mitophagy.^33^, ^38^ Previous studies reported that mitochondria can be directly or indirectly damaged by Cd, which causes excessive PINK1 accumulation on the mitochondrial membrane as a consequence of the inhibition of the respiratory chain. Our present results demonstrate that Cd activated the PINK1/Parkin pathway by promoting the expression of autophagic receptor P62 and NDP52 transported to the defective mitochondria, illustrating the mechanisms underlying the regulation of Cd‐induced mitophagy in neuronal cells. Consistent with our results, Xue and colleagues found that Cd promoted PINK1/Parkin pathway activation in mouse kidneys (Cd induces mitophagy through the ROS‐mediated PINK1/Parkin pathway).

Mitochondria is one of the most important organelles in the regulation of energy generation.^39^ AMPK is a key sensor and regulator of the energy status, and numerous studies have demonstrated that AMPK, when activated by metabolic stress, has the ability to stimulate autophagy by inhibiting mTOR signalling.^40^ Moreover, AMPK‐mediated autophagy is activated by Cd to protect cells from DNA damage.^41^ The decline in levels of AMPKα2, a subunit of AMPK, also correlates with an accumulation of dysfunctional mitochondria, implicating that AMPKα2 is a necessary factor for mitophagy.^42^, ^43^ Knock‐down of AMPK inhibits Beclin1 expression and autophagosome formation, attenuating the enhancement of autophagy in Cd‐exposed mesenchymal stem cells in vitro.^44^ In agreement with previous studies, our results show that Compound C and si‐AMPK inhibited the transportation of Beclin1 to mitochondria. Gelmetti et al found that Beclin1 directly interacts with PINK1 at mitochondrion‐associated membranes, which is critical for mitophagosome formation.^45^ Moreover, AMPKα2 was shown to interact with PINK1 in mitophagy initiation.^32^ Here, we found that pharmacological blockade of AMPK activation with Compound C can attenuate the observed mitophagy while inhibiting Cd‐induced PINK1 expression in PC12 cells. Collectively, these results demonstrate that PINK1 is a possible target of AMPK to assemble mitochondria into autophagosomes. However, the relationship between AMPK and the PINK1/Parkin pathway in Cd‐induced mitophagy and neurotoxicology requires further clarification.

In conclusion, our results suggest that Cd triggers mitophagy in a PINK1/Parkin‐dependent way via AMPK in PC12 cells. In particular, our data demonstrate that PINK1‐dependent mitochondrial degradation may play a critical role in the protecting PC12 cells from Cd‐induced inflammation. These results contribute to our understanding of Cd‐mediated neurotoxicology.

## CONFLICT OF INTEREST

All authors declare that they have no conflict of interest.

## AUTHOR CONTRIBUTIONS

ZPL and TW conceived and designed the study. TW, QPZ and BBC performed the experiments. YY contributed materials and methods. SQW analysed the data. TW wrote the manuscript.

## Data Availability

The data that support the findings of this study are available from the corresponding author upon reasonable request.
